# Long-term survival in extensive-stage small cell lung cancer: a case report on the integration of interventional bronchoscopy and systemic immunotherapy

**DOI:** 10.3389/fonc.2025.1733112

**Published:** 2026-01-20

**Authors:** Ganxiu Deng, Shixuan Yan, Victor Bwembya, Xiaoping Zhu, Zhibing Luo, Changwen Deng

**Affiliations:** 1 Department of Respiratory and Critical Care Medicine, Shanghai East Hospital, School of Medicine, Tongji University, Shanghai, China; 2 Department of Pulmonary and Critical Care Medicine, Punan Hospital, Pudong New District, Shanghai, China; 3 Shenzhen Ruipuxun Academy for Stem Cell & Regenerative Medicine, Shenzhen, China

**Keywords:** case report, central airway obstruction, cryo-immunotherapy, interventional pulmonology, small-cell lung cancer, tumor microenvironment

## Abstract

Small cell lung cancer (SCLC) is an aggressive malignancy with a poor prognosis, particularly in patients with extensive-stage disease (ES-SCLC). Although the incorporation of immune checkpoint inhibitors (ICIs) into first-line chemotherapy has modestly improved survival, long-term disease control remains rare. Central airway obstruction (CAO), a common complication of advanced SCLC, often leads to respiratory failure and interruption of systemic therapy, further compromising outcomes. We report the case of a 65-year-old man diagnosed with ES-SCLC based on endobronchial ultrasound-guided transbronchial needle aspiration. The patient initially responded to platinum–etoposide chemotherapy but experienced treatment interruption during the COVID-19 pandemic, followed by recurrent malignant airway obstruction and respiratory failure. Repeated bronchoscopic interventions, including tumor debulking, airway stent placement, electrocautery, laser therapy, and cryotherapy, were performed to restore airway patency and stabilize respiratory function, thereby enabling the resumption and continuation of systemic antitumor therapy. Subsequently, chemotherapy combined with the programmed cell death protein-1 (PD-1) inhibitor serplulimab was initiated, followed by maintenance immunotherapy. After local bronchoscopic ablation, transient elevations in circulating inflammatory cytokines, including interleukin-6 and interleukin-8, were observed, suggesting systemic immune activation. The patient achieved sustained partial remission, with preserved airway patency and good general condition. At five years after initial diagnosis, the patient remains alive with stable disease and without severe treatment-related adverse events. This case highlights the potential role of integrating bronchoscopic local interventions with systemic chemotherapy and immunotherapy to enable durable disease control and long-term survival in selected patients with ES-SCLC complicated by central airway obstruction.

## 1 Introduction

Small cell lung cancer (SCLC) is an aggressive neuroendocrine malignancy characterized by rapid tumor growth, early metastatic dissemination, and a high rate of recurrence (1). Approximately 10–15% of all lung cancers are classified as SCLC, and nearly 70% of patients present with extensive-stage disease (ES-SCLC) at the time of diagnosis (2–4). Despite initial sensitivity to platinum–etoposide chemotherapy, the majority of patients experience early disease progression, resulting in poor long-term outcomes. In recent years, the addition of immune checkpoint inhibitors (ICIs) to first-line chemotherapy has modestly improved overall survival in ES-SCLC (5, 6). however, durable disease control and long-term survival remain uncommon and are typically observed only in a small subset of patients (7–10).

Due to its rapid growth pattern and tendency for peribronchial infiltration, SCLC frequently involves the central airway, leading to central airway obstruction (CAO) in patients with advanced disease (4). CAO can cause severe dyspnea, post-obstructive pneumonia, and respiratory failure, often resulting in deterioration of performance status and interruption or discontinuation of systemic antitumor therapy. While chemotherapy and radiotherapy remain the cornerstone of treatment for ES-SCLC, these modalities are often insufficient to rapidly relieve life-threatening airway compromise (11). In this setting, bronchoscopic interventions—including tumor debulking, airway stent placement, and local ablation techniques—play a critical role in restoring airway patency and stabilizing respiratory function. Traditionally regarded as palliative measures, these interventions have primarily been used to alleviate symptoms and improve quality of life in patients with malignant airway obstruction (12, 13).

Emerging evidence suggests that local tumor ablation may exert immunomodulatory effects beyond mechanical airway relief by inducing tumor antigen release and remodeling the tumor microenvironment toward a more immunologically active state (14–19). This has generated growing interest in the potential synergy between local ablative therapies and systemic immunotherapy. However, clinical evidence supporting the integration of bronchoscopic local interventions with immune checkpoint blockade in ES-SCLC remains limited. In this context, we report a rare case of ES-SCLC complicated by recurrent central airway obstruction, in which repeated bronchoscopic interventions combined with chemotherapy and immunotherapy were associated with sustained disease control and long-term survival.

## 2 Case description

**Figure 1:**
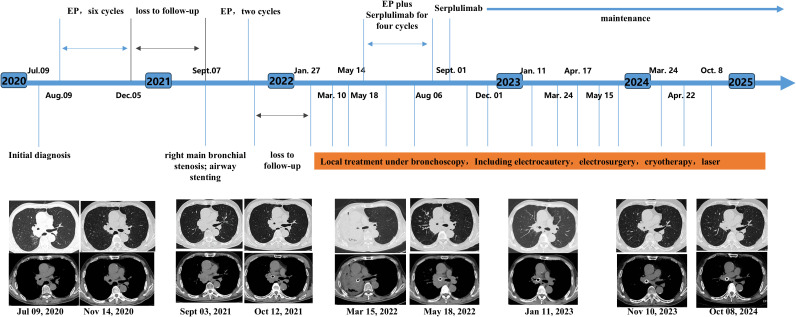
Timeline of patient treatment. EP, etoposide plus cisplatin chemotherapy regimen.

A 65-year-old male was admitted to Shanghai East Hospital in July 2020 after chest computed tomography (CT) revealed a mass in the right hilar region accompanied by multiple enlarged mediastinal lymph nodes (**Figure 1**). The patient had a 30-year history of cigarette smoking (approximately one pack per day). Endobronchial ultrasound-guided transbronchial needle aspiration (EBUS-TBNA) of the right hilar lesion was performed, and pathological examination confirmed small cell lung cancer (SCLC). Immunohistochemical analysis demonstrated positivity for CD56, chromogranin A, and synaptophysin, with a Ki-67 proliferation index of approximately 60%, while PD-1 and PD-L1 expression were negative. Further evaluation revealed enlarged lymph nodes in the right axilla. Brain magnetic resonance imaging and contrast-enhanced abdominal CT showed no evidence of distant metastases. Based on these findings, the patient was diagnosed with extensive-stage SCLC (ES-SCLC).

After exclusion of treatment contraindications, the patient received six cycles of first-line platinum–etoposide (EP) chemotherapy (etoposide 100 mg/m² on days 1–3 and cisplatin 75 mg/m² on day 1, every 3 weeks) from August to December 2020. A partial radiological response was achieved, with improvement in clinical symptoms. However, regular follow-up and further treatment were interrupted due to the COVID-19 pandemic.

**Figure 2:**
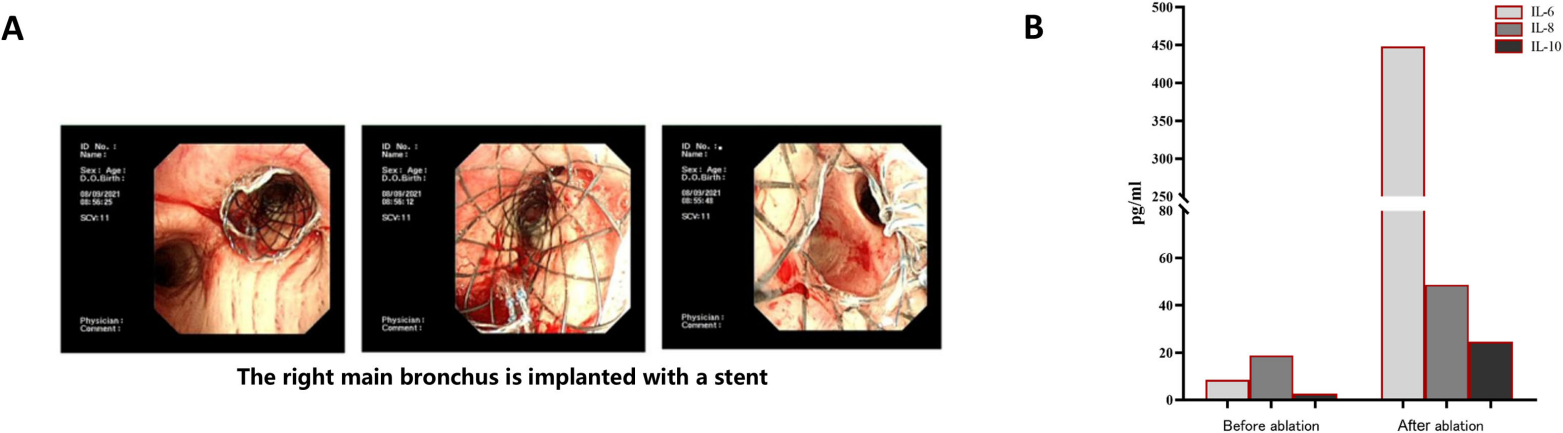
**(A)** The right main bronchus is implanted with a stent under bronchoscopy; **(B)** circulating cytokines IL-6 and IL-8 significantly increased after bronchoscopic local interventional therapy.

In September 2021, the patient was readmitted with severe dyspnea and hypoxemia, with an oxygen saturation of 85% on room air. Chest CT demonstrated severe stenosis of the right main bronchus, which was confirmed by bronchoscopy, showing near-complete luminal obstruction caused by tumor tissue (**Figure 1**). Urgent bronchoscopic tumor debulking was performed, followed by placement of a self-expandable metallic airway stent (**Figure 2A**). After relief of the central airway obstruction, the patient’s respiratory symptoms and oxygenation improved markedly. His overall clinical condition and performance status improved sufficiently to allow stabilization of respiratory failure and resumption of systemic antitumor therapy. Based on the favorable response to prior chemotherapy, the EP regimen was reinitiated on September 14, 2021.

Despite initial clinical stabilization, regular follow-up was again disrupted during the pandemic period. In early 2022, the patient was readmitted because of recurrent dyspnea due to restenosis of the right main bronchus. During this period, multiple bronchoscopic interventions were performed to maintain airway patency, including electrocautery, laser therapy, ablation, and cryotherapy. Cryotherapy was administered using repeated freeze–thaw cycles under bronchoscopic guidance, with each freeze lasting approximately 20–40 seconds, tailored to the extent of endobronchial tumor involvement. No clinically significant complications related to tumor debris retention, massive hemoptysis, or procedure-related infection were observed. Supportive treatment, including anti-infective therapy and oxygen supplementation, was provided as needed.

Following bronchoscopic local ablation, peripheral blood analysis revealed transient elevations in circulating inflammatory cytokines, including interleukin-6 (IL-6) and interleukin-8 (IL-8) (**Figure 2B**). The patient’s respiratory status improved, and airway patency was successfully restored.

Subsequently, systemic therapy was escalated to combination treatment with chemotherapy and the programmed cell death protein-1 (PD-1) inhibitor serplulimab. After four cycles of combination therapy, the treatment regimen was transitioned to maintenance monotherapy with serplulimab. During follow-up, the patient underwent periodic bronchoscopic interventions, including ablation and cryotherapy, to maintain airway patency. No severe immune-related adverse events were observed, and no clinically significant complications related to the airway stent, such as infection, hemoptysis, or symptomatic granulation tissue formation, occurred.

Radiological evaluation demonstrated sustained partial remission, with marked reduction of the right hilar mass and preservation of patency in the right main bronchus. At five years after the initial diagnosis, the patient remains alive with stable disease and good general condition. The detailed clinical course and treatment timeline are summarized in **Table 1**.

**Table 1:** Timeline of clinical course, bronchoscopic interventions, and systemic therapy.

Time	Clinical events	Bronchoscopic intervention	Systemic therapy	Clinical outcome
Jul 2020	Right hilar mass and mediastinal lymphadenopathy detected on chest CT; diagnosis of ES-SCLC confirmed by EBUS-TBNA	None	None	Initial diagnosis established
Aug–Dec 2020	First-line treatment initiated	None	EP chemotherapy (etoposide + cisplatin), 6 cycles	Partial radiological response; symptoms improved
Dec 2020–Sep 2021	Follow-up interrupted due to COVID-19 pandemic	None	Treatment discontinued	Disease progression suspected
Sep 2021	Readmission with severe dyspnea and hypoxemia; severe right main bronchus stenosis on CT and bronchoscopy	Tumor debulking and placement of self-expandable metallic airway stent	EP chemotherapy reinitiated	Rapid relief of airway obstruction and respiratory symptoms
Late 2021	Clinical stabilization	None	Continuation of EP chemotherapy	Temporary disease control
Early 2022	Recurrent airway stenosis with dyspnea	Repeated bronchoscopic interventions including electrocautery, ablation, and cryotherapy	Supportive care	Airway patency restored; respiratory status improved
Early 2022	Post-interventional immune activation observed	—	—	Circulating IL-6 and IL-8 levels significantly increased
2022	Systemic treatment escalation	Repeated bronchoscopic local ablation and cryotherapy as needed	Chemotherapy combined with PD-1 inhibitor (Serplulimab), 4 cycles	Partial remission achieved
2022–2025	Long-term disease control	Periodic bronchoscopic interventions for airway maintenance	Maintenance immunotherapy with Serplulimab	Sustained partial remission; good general condition at 5-year follow-up

## 3 Discussion

Small cell lung cancer (SCLC) is characterized by rapid tumor growth, early metastatic dissemination, and a high propensity for relapse, resulting in a historically poor prognosis, particularly in patients with extensive-stage disease (ES-SCLC) (4). Although the addition of immune checkpoint inhibitors (ICIs) to platinum–etoposide chemotherapy has modestly improved overall survival, durable long-term survival remains uncommon and is typically confined to a small subset of patients (20). In this context, the present case is notable for achieving sustained disease control and survival exceeding five years, highlighting the potential clinical value of integrating bronchoscopic local interventions with systemic chemotherapy and immunotherapy.

Central airway obstruction (CAO) is a frequent and life-threatening complication in advanced SCLC, often leading to respiratory failure, recurrent infections, and rapid deterioration in performance status. While systemic therapy remains the cornerstone of treatment, it is often insufficient to promptly relieve critical airway compromise. In such scenarios, bronchoscopic interventions play a pivotal role by rapidly restoring airway patency, improving ventilation and oxygenation, and stabilizing the patient’s clinical condition (11, 12, 21). In our case, repeated episodes of severe airway stenosis led to respiratory failure and interrupted systemic therapy. Timely bronchoscopic tumor debulking and airway stent placement effectively alleviated obstruction, enabling the patient to recover respiratory function and resume systemic antitumor treatment. Importantly, these interventions did not directly enhance drug delivery but rather improved physiological reserve and treatment tolerance, thereby allowing continued chemotherapy and immunotherapy. Beyond their mechanical and palliative benefits, bronchoscopic local ablation techniques may exert immunomodulatory effects that complement systemic immunotherapy (22, 23). Local tumor ablation induces tumor necrosis and apoptosis, facilitating the release of tumor-associated antigens and danger-associated molecular patterns, which can promote dendritic cell activation and antigen presentation (24, 25). This process may remodel the tumor microenvironment from an immunologically “cold” state toward a more inflamed and immune-responsive phenotype (17, 19, 26). Preclinical and clinical studies have demonstrated that local ablation can increase infiltration of CD4^+^ and CD8^+^ T cells while reducing immunosuppressive populations such as regulatory T cells, thereby enhancing antitumor immune responses. Moreover, ablation-induced cytokine release, including IL-6 and IL-8, has been associated with immune activation and may contribute to systemic antitumor effects. In the present case, circulating levels of IL-6 and IL-8 were markedly elevated following bronchoscopic local ablation, suggesting a transient systemic inflammatory and immune activation response. While causality cannot be definitively established in a single case, this observation raises the possibility that repeated bronchoscopic ablation may have synergized with PD-1 blockade to enhance immune-mediated tumor control. Recent evidence supporting abscopal effects and STING-dependent type I interferon signaling following cryoablation further supports the biological plausibility of combining local ablation with immunotherapy (19). The durable partial remission observed in this patient may reflect not only effective airway management but also a favorable interaction between local tumor destruction and systemic immune modulation.

From a safety perspective, the patient tolerated repeated bronchoscopic interventions and long-term immunotherapy well, without major procedure-related complications, severe immune-related adverse events, or clinically significant stent-related sequelae such as hemoptysis, infection, or symptomatic granulation tissue formation. This favorable safety profile underscores the feasibility of repeated bronchoscopic interventions as part of a multidisciplinary treatment strategy in carefully selected patients.

Nevertheless, this case has several limitations. The patient’s treatment course and follow-up were intermittently disrupted during the COVID-19 pandemic, potentially influencing disease assessment and management continuity. Additionally, although multiple bronchoscopic biopsies were performed, molecular subclassification of SCLC based on transcriptional or immune signatures was not feasible, limiting insights into predictive biomarkers of immunotherapy response. Furthermore, multiple bronchoscopic modalities—including electrocautery, laser therapy, ablation, and cryotherapy—were used in combination, making it difficult to determine which specific technique contributed most significantly to the observed clinical benefit.

In conclusion, this case illustrates that bronchoscopic local interventions can serve not only as life-saving measures for malignant airway obstruction but also as critical enablers of sustained systemic therapy in ES-SCLC. When integrated with chemotherapy and immunotherapy, these interventions may contribute to prolonged disease control and long-term survival in selected patients. Although broader validation is required, this case supports a multidisciplinary treatment paradigm in which local airway management and systemic immunotherapy are strategically combined to optimize outcomes in advanced SCLC.

## Data Availability

The datasets presented in this article are not readily available because of ethical and privacy restrictions. Requests to access the datasets should be directed to the corresponding author/s.
